# 

**Published:** 1902-05

**Authors:** 


### PERSONALS.

Dr. E. M. Ranck, of the Mulford Company Bacteriological Laboratories, Philadelphia, Pa., made a trip through the South in March.

Governor A. B. Cummins, of Iowa, appointed Dr. P. O. Koto, of Forest City, Iowa, State Veterinarian. Dr. Koto is a graduate of the Chicago Veterinary College, and has been in practice a number of years in the "Hawk Eye State," and was President of the State Veterinary Medical Association last year. He is also engaged in the drug business. Dr. Koto succeeds Dr. J. I. Gibson, of Denison.

Dr. C. T. Goentner, of Bryn Mawr, Pa., has recovered from his recent serious illness sufficient to be at his post of duty in practice.

Dr. Wm. Dougherty, of Baltimore, Md., rarely ever misses his annual pilgrimage to New York at the close of the session of his alma mater.

Dr. Leonard Pearson, of Philadelphia, spent a week among the breeding farms of Illinois as a further means of enlightening the breeders of Pennsylvania on the production of better types of horses.

Dr. H. A. Dewey, of Indianapolis, Ind., has just fallen heir to four thousand dollars, left him by a lady whose life he was instrumental in saving a number of years ago.

Dr. Alex. Glass, of Philadelphia, Pa., hailed with joy the warm days and hied himself away to the waters of the Delaware to gratify a long winter's dream of the joys of yachting.

Dr. W. E. A. Wyman, formerly of Milwaukee, Wis., has succeeded to the practice of Dr. J. S. Grant, of Portland, Mich.

Dr. W. Horace Hoskins, of Philadelphia, Pa., was a visitor to the courts of York county in April, directing the prosecution of Dr. C. E. Hyle, charged with violation of the State laws regulating the practice of veterinary science.

Dr. M. E. Knowles, of Helena, Montana, spent a day in Kansas City in March, on his way to Fort Worth, where he attended a Cattleman's Convention. The Doctor's attendance was in the interest of sanitary control of scab in both cattle and sheep.

Joseph J. Kinyoun, M.D., Ph.D., late Surgeon of the Marine Hospital Service and Director of the Hygienic Laboratory at Washington, has assumed the directorship of the biological laboratories of the H. K. Mulford Company, at Glenolden, Pa.

Dr. Kinyoun has not only served the Government by being delegated special representative to the International Congresses, by being late Surgeon of the United States Marine Hospital Service and Director of the Hygienic Laboratory at Washington, but also by representing the government on several occasions in the investigation of the progress of serum organotherapy and infectious diseases at home and abroad, in Paris and Berlin particularly, Europe generally, and in Japan.

Of necessity his work carried him to and in contact with Professors Koch, Roux, Behring and Pasteur, under whom he has received special instructions and enjoyed the advantages of the study of bacteriology and allied subjects; this, together with the natural tendency of his talents, that of original bacteriological research, peculiarly fits him for the office he now assumes.

Dr. J. C. Foelker, of Allentown, Pa., spent a day in Philadelphia in March. An hour at the JOURNAL office made up a part of his stay.

President Winchester, of the A. V. M. A., was at the annual banquet of the New York American Veterinary College on April 1st, at the Hotel Marlborough.

Dr. R. P. Lyman, of Hartford, Conn., was a visitor to the JOURNAL office in April.

Dr. J. W. Scheibler, of Memphis, Tenn., has a lawsuit on his hands relative to the destruction of some tuberculous cows.

Dr. Edward Martin, of the Van Croft Stock Farm, of Wellsburg, West Virginia, was very much interested in the Pittsburg Kennel Club Show.

The Mechano Neural Therapy College and Hospital, at Trenton, under the control and direction of Drs. Harry Walter and Arnold, have closed their doors.

The many friends of Dr. J. C. McNeil, of Pittsburg, will be glad to learn of his convalescence from a severe attack of typhoid fever.

State Veterinarian Pearson was a visitor to Hazleton, investigating an alleged outbreak of glanders among some mules brought from Missouri to one of the mines.

Mrs. H. D. Gill, of New York City, added another laurel to the wreath of victories won by her famous horse "Jack," at Sea Cliff, L. I., in a free-for-all race.

Dr. Ray J. Stanclift, of the Eighth United States Cavalry, has been transferred from Camp R. S. Mackenzie, Cuba, to Fort Reno, Oklahoma, I. T.

Dr. A. T. Sellers, of Camden, N. J., is a fancier of thoroughbred fowls and a constant reader of the poultry and fanciers' journals.

Dr. E. H. Brown, late Veterinary Surgeon of the United States Army, is now located in general practice at Boise, Idaho. Veterinary dentist W. G. M. Allen is associated with him. They jointly conduct a veterinary hospital and centre for mining and prospecting outfits.

